# Non-Targeted Metabolomic Analysis of Ethanol Extract of Propolis and Its Anti-Inflammatory Effects in LPS-Induced BV2 Microglial Cells via the TLR4 Signaling Pathway

**DOI:** 10.3390/nu17172831

**Published:** 2025-08-30

**Authors:** Xiaolan Xu, Chunxia Li, Yuxuan Zhu, Shuangshuang Zhao, Fangjing Wu, Qian He, Lizhen Wei, Xinle Duan, Jianghong Li

**Affiliations:** 1 College of Bee Science and Biomedicine, Fujian Agriculture and Forestry University, Fuzhou 350002, China; xlxufz@126.com (X.X.); 18887266780@139.com (C.L.); 18638694709@163.com (S.Z.); 13088247367@163.com (F.W.); 15086045820@163.com (Q.H.); 18078560363@163.com (L.W.); 2 College of Food science, Fujian Agriculture and Forestry University, Fuzhou 350002, China; yuxuan18350993003@126.com

**Keywords:** propolis, metabolomics, flavonoids, phenols, microglia, anti-inflammatory

## Abstract

Propolis contains abundant flavonoid and phenolic compounds, whose composition and concentration vary significantly in different geographical origins, thereby affecting its bioactive properties including anti-inflammatory, antioxidant, and antimicrobial activities. In this study, the flavonoid and phenolic content in the ethanol extract of propolis (EEP) from Henan (HN) and Shandong (SD) provinces was quantitatively analyzed, and the results showed that concentrations of both bioactive components in HN were slightly higher than those in SD. The non-targeted metabolomics technology was further employed to analyze the components of EEP, and a total of 10683 metabolites were detected. In the comparison between the samples of HN and SD, there were a total of 1436 differential metabolites, with 553 decreased and 883 increased in the HN sample. Among them, there were 205 differential metabolites related to flavonoids and phenols, with 108 decreased and 97 increased in the HN sample. However, a greater number of carboxylic acids and derivatives, fatty derivatives and organooxygen metabolites were found at higher relative levels in the HN sample. As a result, the EEP of the HN sample was selected to investigate its inhibitory effect on inflammation in lipopolysaccharide (LPS)-induced BV2 microglia cells. The results showed that LPS promoted the M1 polarization of BV2 microglia. However, treatment with EEP at concentrations of 10 µg/mL, 5 µg/mL, and 2.5 µg/mL could partially restore the cell morphology to its non-activated state. Meanwhile, LPS stimulation increased the protein levels of IL-1β, IL-6 and TNF-α significantly, as well as the relative gene expression levels of *IL-1β*, *IL-6*, *TNF-α*, *COX-2*, *iNOS* and TLR4. After treatment with the EEP, the expression levels of these three proteins and six genes were significantly decreased. These findings revealed that EEP effectively inhibited the M1 polarization of LPS-induced BV2 cells and decreased the expression of inflammatory factors, indicating its potential as a therapeutic agent for neuroinflammation.

## 1. Introduction

Propolis is a resinous compound collected by honeybees from plant buds and tree bark, mixed with their own saliva and beeswax. It is a nutrient-rich natural substance exhibiting various pharmacological properties, including anti-inflammatory, tumor-suppressing, antimicrobial, and blood sugar-regulating properties [1,2,3]. The components in propolis are very complex, primarily including flavonoids, terpenes, and phenolic acids [4]. Among them, caffeic acid phenethyl ester (CAPE), quercetin, apigenin, ferulic acid, dimethoxy cinnamic acid, isorhamnetin, kaempferol and their derivatives had been confirmed the properties of antimicrobial, antioxidant, antiviral, and anti-inflammatory [5,6].

The ethanol extract of propolis (EEP), whose important components were flavonoids and phenolic constituents, exhibits significant anti-inflammatory activity. These bioactive components can prevent atherosclerosis, modulate blood lipids profiles, regulate angiogenesis, indicating that EEP is a promising therapeutic agent for the prevention of cardiovascular diseases [7]. The anti-inflammatory mechanisms of propolis include inhibiting the production of inflammatory factors, regulating inflammatory signaling pathways, and inhibiting the activation of inflammatory cells. Propolis can inhibit the levels of inflammatory factors such as tumor necrosis factor alpha (TNF-α), interleukin-6 (IL-6), and interleukin-1 beta (IL-1β), thereby reducing the degree of inflammatory response [8,9]. In addition, it can inhibit the expression of inflammation related genes and regulate signaling pathways such as mitogen-activated protein kinase (MAPK) and Toll-like receptor 4 (TLR4)-nuclear factor kappa-B (NF-κB) to suppress the occurrence of inflammatory reactions [10,11]. Some reports showed that the anti-inflammatory effect mainly come from some components in propolis, such as resveratrol, apigenin, caffeic acid, etc., through reducing the expression of inflammatory factors and regulating related signaling pathways [10,11].

BV2 microglial cells are a type of immune effector cell that originate from the brain and spinal cord. Under normal conditions, the BV2 cells exist in a highly branched and resting state, playing a role in dynamically monitoring and responding to the neural environment [12]. When the nervous system is injured or in an inflammatory state, the cells become activated and rapidly differentiate into immune cells with phagocytic functions, engulfing necrotic cells and repairing damaged neural tissue [13]. However, when BV2 cells are over activated, they continuously release a series of inflammatory cytokines, such as IL-1β, IL-6, and TNF-α, leading to various physiological inflammatory complications [14,15]. The activation of BV2 cells is typically considered a pathological feature of Parkinson and Alzheimer diseases, and is closely associated with neurodegenerative disorders. Through the release of inflammatory factors, the activated BV2 can exert neurotoxic effects, resulting in neural damage and dysfunction [16].

Numerous studies have investigated the effects of natural compounds on microglial inflammation [17,18]. Currently, there are few reports on the intervention of EEP in BV2 cell inflammation. In this study, the components of propolis from Henan (HN) and Shandong (SD) provinces in China, were analyzed using non-targeted metabolomics technology, and the protective effect of EEP from HN on lipopolysaccharide (LPS)-induced BV2 cells were determined. This study sought to establish theoretical and experimental basis for propolis as a potential substance for preventing neuroinflammatory diseases.

## 2. Methods

### 2.1. Preparation of EEP

Raw propolis was provided by Fujian Shenfeng Science and Technology Co., Ltd. (Fuzhou, China), and was collected from Kaifeng City, Henan Province and Jining City, Shandong Province, respectively. EEP was prepared using the method described in our previous research [19]. In detail, the propolis was dissolved in 70% ethanol (*v*/*v*) and extracted for 48 h. The mixture was then extracted using ultrasonic method at 40 kHz for 20 min. After centrifugation, the supernatant was filtered, concentrated by rotary evaporation, and freeze-dried. The final extract was stored at 4 °C.

### 2.2. Chemical Analysis for Propolis

#### 2.2.1. The Determination of Total Flavonoid and Phenolic Content

Total flavonoid content analysis was performed according to GB/T 24283-2018 (Chinese national standard for propolis: https://openstd.samr.gov.cn/bzgk/gb/newGbInfo?hcno=6BC12ED9BE0F8C637296AE63B669C1F4, accessed on 10 July 2025). The linear calibration curve (y = 15.625x + 0.0363, R^2^ = 0.9991) was yielded for quantification using rutin as an external standard. Total phenolic content was determined using the Folin–Ciocalteu method. Accurately measured 0, 0.1, 0.2, 0.3, 0.4, 0.5, 0.6, 0.8, 1.0, and 1.2 mL of 0.1 mg/mL gallic acid standard solution into separate volumetric flasks, added 6 mL of distilled water to each flask, and then added 0.5 mL of 2 mol/L phenol reagent and mixed gently. After 1 min, 1.5 mL of 20% sodium carbonate solution was added and the volume was adjusted to 10 mL with sterile water. The absorbance was measured at 765 nm after 10 min, yielding a standard curve of y = 15.625x + 0.0363 with R^2^ = 0.9991.

#### 2.2.2. Non-Targeted Metabolomics Analysis

##### Detection Method of Metabolites

EEP was dissolved with 80% methanol to the concentration of 2 mg/mL. The solution was centrifuged at 4 °C and 13,000 rpm for 10 min. The supernatant was filtered using a 0.22 μm pinhole filter. The metabolic analysis was performed on an ACQUITY UPLC I-Class system (Waters Corporation, Milford, MA, USA) equipped with ACQUITY UPLC HSS T3 (1.8 μm, 2.1 × 100 mm) in both ESI positive and ESI negative ion modes. The binary gradient elution A and B were 0.1% formic acid in water and in acetonitrile, respectively. The gradient was set as follows: 0 min, 5% B; 2 min, 5% B; 4 min, 30% B; 8 min, 50% B; 10 min, 80% B; 14 min, 100% B; 15 min, 100% B; 15.1, 5% B; 16 min, 5% B. The flow rate was 0.35 mL/min and the column temperature was 45 °C. The injection volume was 2 μL. Mass spectrometric data were acquired under full-scan mode (*m*/*z* 100–1200). The parameters were as follows: resolution (full scan), 70,000; Resolution (HCD MS/MS scans), 17,500; spray voltage, 3800 V (+) and 3000 V (−); sheath gas flow rate, 35 arbitrary units; auxiliary gas flowrate, 8 arbitrary units; capillary temperature, 320 °C.

##### Data Preprocessing and Analysis

The original data were processed using Progenesis QI V2.3 software (Nonlinear, Dynamics, Newcastle, UK). The compounds were identified based on precise *m*/*z*, secondary fragments, and isotopic distribution. The compounds were analyzed using HMDB, EMDB, Lipidmaps, PMDB, and Metlin databases. Principle Component Analysis (PCA) was used to observe the stability of the samples, and Orthogonal Partial Least-Squares-Discriminant Analysis (OPLS-DA) was used to distinguish the differential metabolites between various samples. VIP scores derived from the OPLS-DA were employed to rank metabolite contributions to group separation. The statistical significance of intergroup metabolic differences was assessed using two-tailed Student’s *t*-tests. Differential metabolites were identified based on the criteria of VIP >1.0 and *p* < 0.05.

### 2.3. Anti-Inflammatory Assays in BV2 Cells

#### 2.3.1. Cell Recovery and Culture

The cryovial containing BV2 microglial cells, which purchased from Procell Life Science and Technology Co., Ltd. (Wuhan, China), was quickly removed from liquid nitrogen and thawed in a 37 °C water bath. The cell suspension was promptly transferred to a centrifuge tube containing 3 mL of BV2 cell-specific culture medium (Procell Life Science and Technology Co., Ltd., Wuhan, China), and centrifuged at 1000 rpm for 5 min. Then the supernatant was discarded, and the cell pellet was resuspended in 3 mL of fresh BV2 cell-specific culture medium. The cells was then cultured in an incubator with 5% CO_2_ at 37 °C.

#### 2.3.2. EEP Solution Preparation and Cell Treatment

Exactly 0.1 g of EEP was dissolved in 0.5 mL DMSO, then diluted with BV2 cell-specific culture medium to yield a final concentration of 100 µg/mL. LPS (0.1 g) was dissolved in BV2 cell-specific culture medium and then diluted to obtain a stock solution with a concentration of 10 µg/mL for subsequent experiments. The cell concentration was adjusted to 1 × 10^5^ cells/mL and inoculated in a 96-well plate at 37 °C with 5% CO_2_ for 24 h. when the cells had adhered, the culture medium, LPS and different concentration of EEP were added into various tubes to get the following groups: Control group (culture medium), solvent group (culture medium containing 0.05% DMSO), LPS group (1 µg/mL), EEP1 group (10 µg/mL EEP and 1 µg/mL LPS), EEP2 group (5 µg/mL EEP and 1 µg/mL LPS), EEP3 group (2.5 µg/mL EEP and 1 µg/mL LPS). The solvent group contained an equal concentration of DMSO as the EEP groups. All treatment groups were incubated at 37 °C with 5% CO2 for 24 h. After incubation, the morphology of BV2 cells were observed using Ix53 inverted microscope (Olympus Corporation, Kyoto, Japan) at 200× and 100× magnification.

#### 2.3.3. Cell Viability Assessment

The CCK-8 assay kit (Beyotime Biotechnology, Shanghai, China) was used to evaluate the cell viability of LPS- and EEP-treated BV2 cells. After 24 h of treatment, the culture medium was aspirated, and each well received 100 µL of BV2 cell medium and 10 µL of CCK-8 solution. The cells were then incubated at 37 °C in 5% CO_2_ for 1 h. During the incubation period, the tetrazolium salt WST-8 in the reagent was reduced by cellular dehydrogenases to highly water-soluble yellow formazan dye under the action of electron carrier 1-Methoxy PMS. The amount of formazan generated was proportional to the number of viable cells. Absorbance was measured at 450 nm, and the viability was determined using the equation: viability (%) = (ODtest/OD_control_) × 100%.

#### 2.3.4. ELISA Analysis of TNF-α, IL-6 and IL-1β Expression

The concentrations of TNF-α, IL-6, and IL-1β were determined using ELISA kits (Neobioscience, Shenzhen, China). BV2 cells were cultured in 6-well plates until reaching the logarithmic growth phase. The cells were then treated with trypsin and centrifuged at 1000 rpm for 5 min to discard the supernatant. Cell pellets were washed three times with PBS and resuspended to a final concentration of 5 × 10^6^ cells/mL in PBS. Subsequently, the cells were repeatedly frozen and thawed three times using liquid nitrogen (5 min) and a 37 °C water bath (10 min). The standard curves were constructed according to the ELISA kit instructions. The standard curve of IL-1β was described as y = 0.0203x + 0.2198 (R^2^ = 0.9909), the standard curve of IL-6 was described as y = 0.0184x + 0.149 (R^2^ = 0.9939), and the standard curve of TNF-α was described as y = 0.0038x + 0.1986 (R^2^ = 0.9906). The samples were also treated in accordance with the kit instructions. The samples were incubated with the antibody working solution at 37 °C for 1 h. After the wells were washed three times with PBS, the enzyme conjugate working solution was added, and incubation continued at 37 °C in the dark for 15 min. Then the substrate was added, and incubation proceeded at 37 °C for 15 min. Finally, 100 µL of stop solution was added to each well and thoroughly mixed. Absorbance at 450 nm was immediately measured using the microplate reader.

#### 2.3.5. Measurement of Inflammatory Cytokine Gene Expression by RT-qPCR

RT-qPCR was performed to assess the expression of inflammatory cytokine genes in BV2 cells. Primers specific to BV2 cell-related inflammatory cytokine genes were designed using NCBI Primer-BLAST and the primers are listed in Table 1. *β-Actin* was used as the endogenous control gene for normalization of target gene expression levels. A two-step RT-qPCR protocol was employed, with gene transcription levels calculated using the 2^−ΔΔCT^ method. The reaction system was prepared according to the TB Green^®^ Premix Ex Taq™ II kit instructions (Takara Bio, Kyoto, Japan). The amplification program was as follows: 94 °C for 30 s; followed by 40 cycles, with each cycle consisting of 95 °C for 5 s and 60 °C for 30 s. The melt curve analysis was generated at 65 °C (5 s) followed by 95 °C (5 s).

#### 2.3.6. Statistical Analysis

All experimental procedures were conducted with three biological replicates. Statistical analyses were performed using SPSS 20.0 (IBM Corp., Armonk, NY, USA). Results were presented as mean (*n* = 3) ± SEM. For multi-group comparisons, one-way analysis of variance (ANOVA) was conducted, followed by post hoc tests as appropriate. Statistical significance was defined as *p* < 0.05.

## 3. Results

### 3.1. Untargeted Metabolomics Analysis

#### 3.1.1. Metabonomic Profiling of Propolis

The content of total flavonoids was 317.26 ± 2.54 in HN and 291.35 ± 1.63 in SD. The content of total phenols was 383.56 ± 4.28 in HN and was 357.12 ±1.39 in SD. The results showed that the content of total flavonoids and phenols of EEP in HN was slightly higher than that in SD.

**Figure 1:**
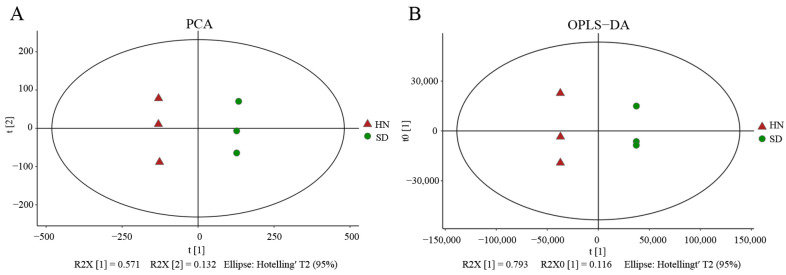
PCA and OPLS-DA analysis of metabolomics data. (**A**) PCA analysis. (**B**) OPLS-DA analysis.

Chemical composition of propolis from the two regions was analyzed using untargeted metabolomics. The results showed that a total of 10,683 metabolites were detected (Supplementary File S1). PCA analysis revealed that the samples from the same region were clustered together, and the samples from two regions were separated. This clustering pattern indicates both high intra-group repeatability and significant inter-regional differences in chemical composition and content (Figure 1A). OPLS-DA analysis further demonstrated the reliability of metabolic data and the differences between the two regions (Figure 1B).

**Figure 2:**
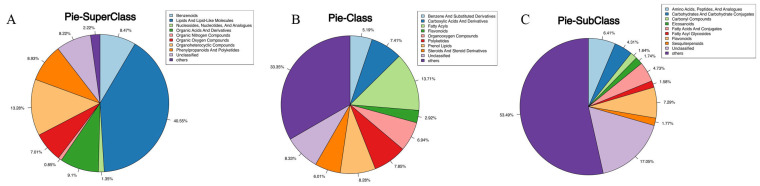
Categorization of all metabolites. (**A**) Superclass categorization. (**B**) Class categorization. (**C**) Subclass categorization.

These 10,683 metabolites were classified at the super class, class and subclass levels. The super class categories included lipids and lipid-like molecules (40.55%), organoheterocyclic compounds (13.28%), organic acids and derivatives (9.10%), phenylpropanoids and polyketides (8.93%), and benzenoids (8.47%), and others (Figure 2A). The class categories included fatty acyls (13.71%), prenol lipids (8.28%), polyketides (7.85%), carboxylic acids and derivatives (7.41%), organooxygen compounds (6.94%), and steroids and steroid derivatives (6.01%), and others (Figure 2B). The sub class categories included flavonoids (7.29%), amino acids, peptides, and analogues (6.41%), fatty acids and conjugates (4.73%), and carbohydrates and carbohydrate conjugates (4.31%), and others (Figure 2C). In the HN-vs.-SD comparison, there were 1436 differential metabolites, among which 883 were increased and 553 were decreased in HN sample (Supplementary File S2).

#### 3.1.2. Analysis of Differential Metabolites of Flavonoids and Phenols

Flavonoids and phenols, along with their derivatives, were the most important bioactive components in propolis. A total of 1417 flavonoid- and phenols-related metabolites were detected in propolis from two regions, primarily including flavones, isoflavones, flavonols, flavanones, flavanols, anthocyanins, chalcones, dihydroflavones, phenols, and phenolic esters.

A total of 205 differential metabolites related to flavonoids and phenols were identified in the HN-vs.-SD comparison (Supplementary File S2). The Flavonoids category has the highest number of differential metabolites (number: 126), while other categories contained fewer, such as flavonoid glycosides (number: 13), O-methylated flavonoids and iso-flavonoids (number: 11), flavones (number: 8), furano-isoflavonoids (number: 2), and pyrano-flavonoids (number: 2). Among these compounds, 97 metabolites were increased and 100 were decreased in the HN sample. Cupressuflavone (log_2_(FC) value: 42.98) showed the most significant fold difference, followed by Epimedoside D (log_2_(FC) value: 39.31). Among the differential metabolites, the important flavonoid compounds included derivatives of quercetin, isorhamnetin, kaempferol, and Pinocembrin (Table 2). In addition, the important phenolic acids were identified, including derivatives of cinnamic, caffeic, quinic acid, coumaric acid, and ferulic acid (Supplementary File S1). Among them, caffeic acid and quinic acid showed significant differences (Table 2).

#### 3.1.3. Other Differential Metabolites Between HN and SD

Terpenes are one of the main active ingredients in propolis. A total of 712 terpenoid-related compounds were detected in propolis from the two different regions. A total of 103 differential metabolites, including 17 diterpenoids, 7 monoterpenoids, 25 sesquiterpenoids, 13 terpene glycosides, 9 terpene lactones, 17 triterpenoids, and 15 isoprenoids. In the HN sample, 54 metabolites were increased and 49 were decreased compared to the SD sample. The terpenes metabolite with the most significant difference was Neopetasin (log_2_ (FC): 40.80) (Supplementary File S2).

From the above analysis, it can be seen that compared to the SD sample, the number of flavonoids, phenols, and terpenes that decreased in the HN sample not significantly different from the number that increased. However, in the HN sample, most of the carboxylic acids and derivatives, fatty acids, and organooxygen metabolites were increased. For example, among amino acids, peptides, and analogues, 46 metabolites were increased and 16 metabolites were decreased. Among fatty acids and conjugates compounds, 68 were increased and 30 were decreased. Among carbohydrates and carbohydrate conjugates, 55 were increased and 12 were decreased. In addition, further analysis revealed that among the 1436 metabolites, 243 metabolites had a log_2_(FC) value greater than 3, while only 8 metabolites exhibited a log_2_(FC) value less than −3 in the HN_vs._SD comparison.

### 3.2. The Anti-Inflammatory Effects of Propolis on LPS-Stimulated BV2 Cells

#### 3.2.1. Effects of EEP and LPS on BV2 Cells Viability

**Figure 3:**
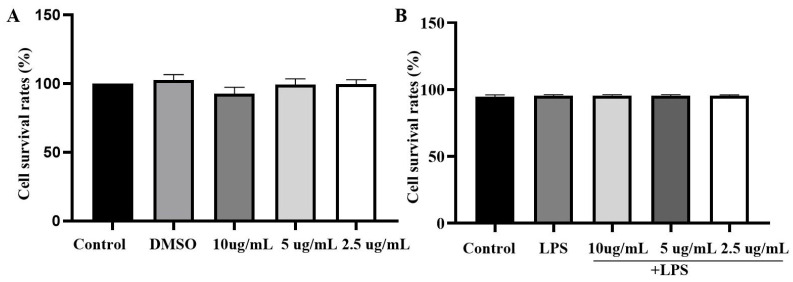
The effects of EEP on BV2 cell activity. **(A**) The effect of EEP on none-LPS treatment of BV2 cell activity; (**B**) the effect of EEP on LPS treatment of BV2 cell activity.

The effects of LPS and EEP on the viability of BV2 microglial cells were determined. The results showed that neither the DMSO nor the propolis treatments (10, 5, and 2.5 μg/mL) caused significant differences in BV2 cell viability compared to the control group (Figure 3A). The effects of the EEP at concentrations of 10, 5, and 2.5 μg/mL on the viability of LPS-treated BV2 cells were shown in Figure 3B. There were also no statistically significant differences in BV2 cell viability among the control group, LPS group and EEP-treated groups (Figure 3B). The results revealed that these concentrations of EEP and LPS did not harm BV2 cells and could be used in subsequent experiments.

**Figure 4:**
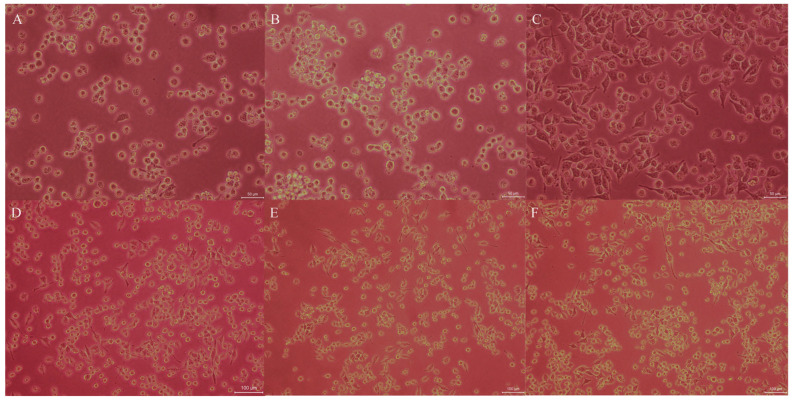
Influence of EEP and LPS on the morphology of Microglia. (**A**) The control group (200×); (**B**) DMSO group (200×); (**C**) LP-induced group (200×); (**D**) 10 μg/mL of EEP group (100×); (**E**) 5 μg/mL of EEP group (100×); (**F**) 2.5 μg/mL of EEP group (100×).

BV2 cells morphology was observed under an inverted microscope, and the results are shown in Figure 4. To better visualize BV2 cells morphology, the control group, DMSO group, and LPS- induced group were observed at 200× magnification. Meanwhile, to more clearly demonstrate the effect of EEP on BV2 cells morphology, the EEP-treated groups were observed at 100× magnification. The cells of the control group exhibited small cells bodies and small branches (Figure 4A). The cells in the 0.05% DMSO group had a morphology similar to that of the control group, indicating that 0.05% DMSO did not affect cell morphology (Figure 4B). LPS-induced cells showed enlarged cells bodies, spindle-shaped protrusions extending toward the poles (Figure 4C). The shape of the cells changed, and the surface of protrusions became thicker, exhibiting an “amoeboid” appearance, indicating that BV2 cells stimulated by LPS had transformed into the activated M1 state, which has phagocytic function. After treatment with 10 μg/mL (Figure 4D), 5 μg/mL (Figure 4E), and 2.5 μg/mL (Figure 4F) of EEP, respectively, the morphology of partial BV2 cells returned to their inactive state, especially the cells in the 10 μg/mL group. This suggested that EEP could partially inhibit the LPS-induced transition of BV2 cells to M1 phenotype, exerting an inhibitory effect on the activation of BV2 cells.

#### 3.2.2. Effects of EEP on the Levels of TNF-α, IL–6 and IL-1β

**Figure 5:**
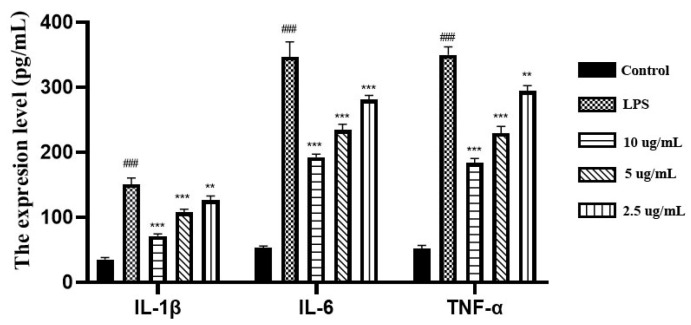
Effects of EEP on the levels of IL-1β, IL-6 and TNF-α in BV2 cells. ^###^ indicates extremely statistically significant differences compared to the control group (*p* < 0.001), *** indicates extremely statistically significant differences (*p* < 0.001) and ** indicates statistically significant differences (*p* < 0.01) compared to the LPS-stimulated group.

ELISA method was used to determine the effects of EEP on the levels of IL-1β, IL-6, and TNF-α in BV2 cells (Figure 5). In the control group, the levels of IL-1β, IL-6, and TNF-α in BV2 cells were very low. After LPS stimulation, the expression levels of these proteins in BV2 cells were significantly increased, indicating that LPS stimulation induced the expression of inflammatory factors in BV2 cells. EEP treatment demonstrated a concentration-dependent suppression of LPS-induced proinflammatory cytokines, with protein levels of IL-1β, IL-6, and TNF-α showing a significant reduction compared to LPS-stimulated controls, with the maximal inhibitory effect observed at 10 μg/mL of EEP.

#### 3.2.3. Effects of EEP on the Expression of Inflammatory Factors

**Figure 6:**
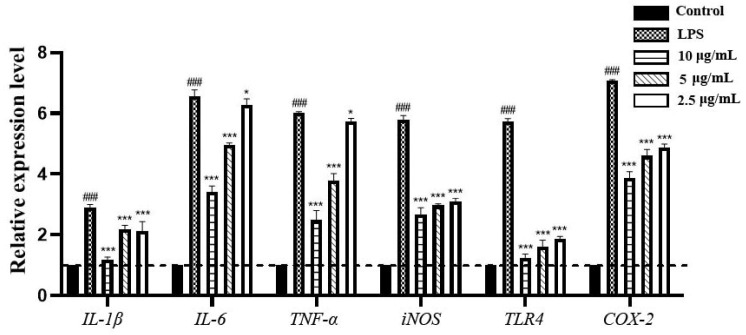
The effect of EEP on the relative expression of inflammatory cytokine factors gene expression in BV2 cells. ^###^ indicates extremely statistically significant differences compared to the control group (*p* < 0.001), *** indicates extremely statistically significant differences (*p* < 0.001) and * indicates statistically significant differences (*p* < 0.05) compared to the LPS-stimulated group.

In this study, RT-qPCR was employed to assess the relative gene expression levels of inflammatory cytokines including *IL-1β*, *IL-6*, *TNF-α*, *iNOS*, *TLR4*, and *COX-2* in BV2 cells. Under the stimulation of LPS, the relative expression levels of these six inflammatory genes were upregulated (Figure 6). After treatment with 10 μg/mL, 5 μg/mL, and 2.5 μg/mL EEP, respectively, the relative expression levels of these genes were downregulated, indicating the significant inhibitory effects of EEP on these inflammatory cytokines. Among them, EEP at a concentration of 10 μg/mL showed the most pronounced inhibitory effect. The relative expression trends of these inflammatory cytokine genes were consistent with their corresponding protein expression trends determined by the ELISA analyses.

## 4. Discussion

Propolis has a complex chemical composition that can vary due to factors like geographical origin, plant sources, and seasons, resulting in differences in the composition and biological activity of propolis from different sources [20,21,22,23]. The main producing regions of propolis include China, Brazil, the United States, Turkey, etc. Most of the propolis produced in China belongs to poplar-type. Its primary chemical constituents are flavonoids, phenolic acids, and terpenoids, among which the compounds with higher content were pinocembrin, pinobanksin, chrysin, p-coumaric acid, caffeic acid and their derivatives [19]. These compounds contribute to the anti-inflammatory effects of propolis while also mediating its antioxidant activity. For example, chrysin, pinocembrin, galangin, and pinobanksin may exert antioxidant effects at low concentration and anti-inflammatory effects at high concentration by regulating the Nrf2 and NF-B pathways [24].

In this study, the flavonoids in propolis included flavans, flavones, flavonoid glycosides, and O-methylated flavonoids. Phenolic acids were mainly composed of caffeic acid, caffeic acid, ferulic acid, and their derivatives. The content of epimedoside ((log_2_(FC) value: 39.31) and cupressuflavone ((log_2_(FC) value: 42.98) in the HN sample was significantly higher than that in SD sample. Both compounds have anti-inflammatory effects, which can enhance antioxidant capacity or reduce the expression of TNF-α, IL-1β, and IL-6 inflammatory factors [25,26]. In this study, the phenolic acids were mainly caffeic acid, ferulic acid, and their derivatives. And these compounds have strong anti-inflammatory activity. For example, CAPE is a potent NF-κB inhibitor that exerts anti-inflammatory effects by inhibiting the NF-κB signaling pathway [27]. In addition, we compared the total content of flavonoids and phenolic substances in two production areas and found that the content of these two types of components in the HN sample was slightly higher than that in the SD samples. Furthermore, compared to the SD sample, the contents of most of the carboxylic acids and derivatives, fatty acids, and organooxygen metabolites were increased in HN sample. Therefore, the HN sample were selected for further anti-inflammatory analysis on microglia.

At present, there are many reports on the types and pharmacological activities of flavonoids and phenolic compounds in propolis [7]. In this study, in addition to these two types of components, a large number of terpenoids were also identified. But the functional properties of terpenoids in propolis remains limited. Previous studies have identified that some substances with similar structures, such as linalool, α-terpineol, α-campholenic acid, and citronellic acid were related to anti-inflammatory effects [28,29]. The sesquiterpenoid nerolidol, identified in propolis essential oil, was found to attenuate the responses of human neutrophil to inflammatory chemoattractants [30]. Previous studies have shown that the anti-inflammatory activity of propolis is related to its flavonoids and phenolic substances [31,32,33]. However, the role, especially the anti-inflammatory activity of propolis terpenoids, deserves further investigation.

BV2 microglia are a type of immune cell and constitute an essential component of the central nervous system (CNS) [34]. CNS injury or inflation rapidly activates microglia, resulting in morphology changes and their polarization into the M1 phenotype with cytotoxicity and the M2 phenotype with neuroprotective effects. LPS or interferon-γ is common substance that induces microglia to switch to the M1 phenotype [35]. M1 microglia produce pro-inflammatory cytokines such as IL-1β, TNF-α, and IL-6, which trigger the inflammatory response and cause damage such as mitochondrial oxidative phosphorylation, lactic acidosis, and excessive accumulation of free radicals, leading to neuronal necrosis [36,37,38]. CAPE in propolis could modulate microglial M1/M2 polarization through the Sirt6/Nrf2 signaling pathway, thereby suppressing oxidative stress [39]. In this study, the results also revealed that the EEP could effectively suppress M1 polarization of BV2 microglia and inhibit the transformation of microglial cells into macrophage-like forms.

IL-1β, TNF-α, and IL-6 are common cytokines. After binding to the receptor, TNF-α can activate the cascade-dependent signaling caspase and TLR signaling pathway [38]. TLR4, as a key receptor, can recognize LPS and initiate immune responses, promote the expression of factors such as TNF-α, and exacerbate inflammation [40,41,42]. The flavonoids in propolis could block the activation of TLR4 and inhibit the nuclear translocation of NF-κB, thereby downregulating the expression of pro-inflammatory factors such as TNF-α, IL-6, and IL-1β [43]. The phenolic compounds in propolis exhibit similar effects. For instance, quercetin in propolis can enhance the antioxidant capacity of LPS-induced BV2 microglial cells, thereby inhibiting their inflammatory response [44]. Propolis and its phenolic substance CAPE can inhibit the proliferation of cancer MDA-MB-231 cells in the inflammatory microenvironment by suppressing the TLR4 signaling pathway [11]. Our previous study demonstrated that Brazilian propolis reduced the levels of inflammatory factors including IL-6, TNF-α, VCAM-1, ICAM-1, and MCP-1 in LPS-induced mouse aortic endothelial cells, thereby exerting anti-inflammatory effects [19]. This study further validated the inhibitory effect of EEP on the expression levels of IL-1 β, TNF-α, IL-6, iNOS, COX-2, and TLR4 in LPS- induced BV2 microglia, thus exerting anti-inflammatory effects.

In summary, this study investigated the compositional profiles of EEP from Henan and Shandong provinces, and analyzed the anti-inflammatory effects of the HN sample on LPS-induced BV2 cells. However, there are certain limitations in this study that require further experimental validation. Firstly, although differences in the composition of EEP between the two regions were revealed, the study did not thoroughly explore how these compositional variations influence anti-inflammatory activity, nor did it determine whether the effects are attributable to specific individual compounds or synergistic interactions among multiple components. Secondly, the conclusions are based exclusively on in vitro experiments and lack validation from in vivo studies. Although propolis was found to inhibit the expression of key genes in the TLR4 pathway in LPS-induced BV2 cells, the inflammatory network in vivo constitutes a highly complex system [45], making it difficult to evaluate the true role of propolis on microglia in vivo. Therefore, identifying the key anti-inflammatory components or component combinations that act on microglial cells, along with elucidating their mechanisms of action, requires further in vitro and in vivo experiments.

## 5. Conclusions

In this study, the total flavonoid and total phenolic content in the EEP from Henan and Shandong provinces were quantitatively analyzed, and the differential metabolites between these samples were identified using non-targeted metabolomics technology. The results revealed that 1436 differential metabolites were identified. Among them, 553 metabolites were decreased and 883 were increased in the HN sample. And the HN sample contained higher levels of total flavonoids and total phenols than the SD sample. Moreover, this study further confirmed that EEP could block the activation of TLR4, thereby downregulating the expression of pro-inflammatory factors (TNF-α, IL-6, and IL-1β) and the production of iNOS and COX-2, consequently inhibiting microglial M1 polarization.

## Figures and Tables

**Table 1:** Primers of RT-PCR.

Primers	Sequences (5′→3′)
*IL-1β*-Forward	GCATGGATCAGAAACTCAGCAA
*IL-1β*-Reverse	TTGAGAGGTGGTGTAAGCCAT
*IL-6*-Forward	GACTGATGCTGGTGACAACC
*IL-6*-Reverse	CAGGTCTGTTGGGAGTGGT
*TNF*-α-Forward	AAGCCTGTAGCCCACGTCGTA
*TNF*-α-Reverse	GGCACCACTAGTTGGTTGTCTTTG
*iNOS*-Forward	ACACCGTGACCGTTGACTAC
*iNOS*-Reverse	CTTCTGCCGGACTTTGGAGT
*TLR4*-Forward	TCTGGGGAGGCACATCTTCT
*TLR4*-Reverse	AGGTCCAAGTTGCCGTTTCT
*COX-2*-Forward	AGGTCATTGGTGGAGAGGTG
*COX-2*-Reverse	CCTGCTTGAGTATGTCGCAC
*β-ACT*-Forward	GGACTGTTACTGAGCTGCGTT
*β-ACT*-Reverse	CGCCTTCACCGTTCCAGTT

**Table 2:** Partially important flavonoids and phenolic acid compounds.

ID	Metabolites	*m*/*z*	Retention Time (min)	Formula	log_2_(FC)	FC
1	Isorhamnetin	315.051	6.8705	C_16_H_12_O_7_	0.0950	1.0681
2	Isorhamnetin 3-(6″-acetylgalactoside)	559.084	4.7782	C_24_H_24_O_13_	7.1654	143.558
3	Dihydroisorhamnetin	317.067	5.1625	C_16_H_14_O_7_	0.5268	1.4407
4	Kaempferol	285.041	6.6709	C_15_H_10_O_6_	0.1172	1.0846
5	8-C-Methylvellokaempferol 3,5-dimethyl ether	412.175	7.6226	C_23_H_22_O_6_	−1.8914	0.2695
6	Kaempferol 3-arabinofuranoside 7-rhamnofuranoside	565.154	5.3921	C_26_H_28_O_14_	−0.3176	0.8023
7	6″,6″-Dimethylpyraono [2″,3″:7,8] kaempferol 4′-methyl ether 3-rhamnoside	495.164	6.7044	C_27_H_28_O_10_	−0.0741	0.9499
8	Kaempferol 3-apiosyl-(1->4)-rhamnoside-7-rhamnoside	755.205	4.557	C_32_H_38_O_18_	3.7788	13.726
9	6-Hydroxyluteolin 7-rhamnoside	429.083	5.3897	C_21_H_20_O_11_	2.6331	6.2038
10	Pinocembrin 7-rhamnosylglucoside	587.173	5.9783	C_27_H_32_O_13_	−0.2939	0.8157
11	Pinocembrin	301.072	6.8534	C_15_H_12_O_4_	0.2451	1.1852
20	5,8-Dihydroxy-7-methoxyflavanone	269.08	10.301	C_16_H_14_O_5_	0.0678	1.0481
21	(2S)-4′,5-Dihydroxy-8-hydroxymethyl-6″,6″-dimethylpyrano 2″,3″:7,6] flavanone	391.117	8.6383	C_21_H_20_O_6_	0.2282	1.1714
22	5,7-Dihydroxy-4′-methoxy-8-C-(2-hydroxy-3-methyl-3-butenyl) flavanone	369.135	9.7757	C_21_H_22_O_6_	0.1829	1.1351
23	5,7,8-Trihydroxy-4′-methoxyflavanone	301.072	7.1025	C_16_H_14_O_6_	−0.7465	0.5960
24	5-Hydroxy-7,8-dimethoxyflavanone	283.096	8.6535	C_17_H_16_O_5_	0.2229	1.1671
25	Glyflavanone A	389.138	9.9047	C_22_H_22_O_5_	0.0632	1.0448
26	Sophoraisoflavanone C	477.263	11.280	C_30_H_36_O_5_	−0.1504	0.9010
27	Lumaflavanone C	489.227	11.556	C_30_H_34_O_7_	0.1278	1.0926
28	7-Hydroxy-6,8-di-C-methylflavanone 7-O-arabinoside	423.143	6.8422	C_22_H_24_O_7_	0.1229	1.0889
29	Glicoisoflavanone	407.147	10.194	C_22_H_24_O_6_	−0.1069	0.9285
30	Epilumaflavanone A	533.218	9.7602	C_30_H_32_O_6_	−1.1109	0.4630
31	Citflavanone	356.149	4.7435	C_20_H_18_O_5_	−1.088	0.4704
32	Prenyl cis-caffeate	247.098	8.3515	C_14_H_16_O_4_	0.1074	1.0773
33	Caffeic acid ethyl ester	209.081	5.6655	C_11_H_12_O_4_	−0.2729	0.8276
34	Caffeic acid	163.039	4.2200	C_9_H_8_O_4_	−1.2746	0.4133
35	Ferulic acid	177.054	4.9568	C_10_H_10_O_4_	−0.3795	0.7687

FC: The ratio of the average expression levels of metabolites in HN_vs._SD comparison. Log_2_(FC): This is the log_2_ logarithmic value of FC. The positive values indicate an increase in content in HN sample and negative values indicate a decrease.

## Data Availability

The data presented in this study are available in Supplementary Files S1 and S2.

## Supplementary Materials

The following supporting information can be downloaded at: https://www.mdpi.com/article/10.3390/nu17172831/s1, Supplementary File S1. All metabolites in the samples of HN and SD; Supplementary File S2. Differential metabolites between the samples of HN and SD.
